# American Medical Association

**Published:** 1902-01

**Authors:** 


### American Medical Association.

New York, November 22, 1901.

*Dr. F. E. Daniel, Austin, Texas.*

Dear Doctor: I have just returned from Saratoga, where the next annual session of the American Medical Association is to be held, having gone there in company with Dr. George H. Simmons, the secretary, and Dr. G. F. Comstock, the chairman of the Committee of Arrangements, to secure accommodations for the various sections. We have secured fourteen halls for the meetings, and have been very fortunate in having these various places within a radius of a quarter of a mile. The Association has never yet met in a place so admirably adapted to its work as Saratoga. The hotels are large enough to comfortably accommodate the entire Association should it attend, and I hope that you and the other good friends of an organized profession throughout the "Lone Star State" will lend their best aid to make this meeting the most largely attended in the history of the Association.

Kindest wishes. Sincerely yours,

JOHN A. WYETH.

---

COLUMBUS, TEXAS, December 5, 1901.

*F. E. Daniel, M. D., Editor Texas Medical Journal, Austin, Texas.*

DEAR DOCTOR: The chairman of the Committee of Ways and Means has appointed me to solicit contributions for the entertainment of medical officers of the army and navy of the Confederacy, the reunion of which is to be held in Dallas April 22, 23 and 24, next. Will you kindly make mention of the fact in the next issue of the JOURNAL, and say that the profession of Texas must acquit itself creditably on this occasion, and anything else you please.

Yours truly,

R. H. HARRISON, M. D.

